# Patch Size and Isolation Predict Plant Species Density in a Naturally Fragmented Forest

**DOI:** 10.1371/journal.pone.0111742

**Published:** 2014-10-27

**Authors:** Miguel A. Munguía-Rosas, Salvador Montiel

**Affiliations:** Departamento de Ecología Humana, Centro de Investigación y de Estudios Avanzados del Instituto Politécnico Nacional (CINVESTAV), Mérida, Yucatán, México; University of Saskatchewan, Canada

## Abstract

Studies of the effects of patch size and isolation on plant species density have yielded contrasting results. However, much of the available evidence comes from relatively recent anthropogenic forest fragments which have not reached equilibrium between extinction and immigration. This is a critical issue because the theory clearly states that only when equilibrium has been reached can the number of species be accurately predicted by habitat size and isolation. Therefore, species density could be better predicted by patch size and isolation in an ecosystem that has been fragmented for a very long time. We tested whether patch area, isolation and other spatial variables explain variation among forest patches in plant species density in an ecosystem where the forest has been naturally fragmented for long periods of time on a geological scale. Our main predictions were that plant species density will be positively correlated with patch size, and negatively correlated with isolation (distance to the nearest patch, connectivity, and distance to the continuous forest). We surveyed the vascular flora (except lianas and epiphytes) of 19 forest patches using five belt transects (50×4 m each) per patch (area sampled per patch = 0.1 ha). As predicted, plant species density was positively associated (logarithmically) with patch size and negatively associated (linearly) with patch isolation (distance to the nearest patch). Other spatial variables such as patch elevation and perimeter, did not explain among-patch variability in plant species density. The power of patch area and isolation as predictors of plant species density was moderate (together they explain 43% of the variation), however, a larger sample size may improve the explanatory power of these variables. Patch size and isolation may be suitable predictors of long-term plant species density in terrestrial ecosystems that are naturally and anthropogenically fragmented.

## Introduction

MacArthur and Wilson’s Equilibrium Theory of Island Biogeography (ETIB) postulates that the number of species in oceanic islands can be predicted by island size and isolation. This is firstly because of sample area effects (in any region, larger sample areas will contain more species than smaller samples) and secondly, but more importantly, because of the interplay between immigration and extinction that leads, over time, to a size and isolation-dependent equilibrium number of species (species relaxation) [1], [2]. Therefore, the main testable predictions of this theory are that the larger and less isolated the island, the higher the species number at which it should reach equilibrium. The ETIB reached paradigmatic status in biogeography and ecology, and also had an enormous impact on conservation biology [3]. The ETIB has also provided a theoretical framework for understanding habitat fragmentation and making predictions about its effect on biodiversity [3], [4], [5], [6], [7].

Since the publication of original monograph of the ETIB in 1967 [2], the authors themselves have suggested that the principles and predictions of the ETIB may apply not only to oceanic islands but also to terrestrial ecosystems that are naturally and anthropogenically fragmented ([2], pp 3–4). However, the current literature is divided about the effects of patch size and isolation on species richness and other measures of biodiversity, such as species density [8], [9], [10], in terrestrial ecosystems [7], [11], [12]. In fact, quantitative reviews have shown that patch area and isolation alone are poor predictors of species occupancy in terrestrial forest fragments [13], [14]. While the low predictive power of patch area and isolation for biodiversity could be due to several confounding factors associated with anthropogenic disturbance (i.e., patches differ in habitat matrix, elevation, human activity, etc. [15], [16], [17]), experimental studies in which these factors are controlled have also shown a remarkable lack of consistency in their results, especially with regard to number of species relative to patch size [18], [19], [20]. A basic condition of the ETIB is that it is only when colonization and emigration rates have reached equilibrium that species number can be accurately predicted by habitat size and isolation [1], [2]. Owing to the long life cycle of some plants [21], it may take hundreds or thousands of years after fragmentation to reach a new equilibrium in forest patches [22], [23]. Studies addressing the effect of patch and landscape traits on plant biodiversity in anthropogenic forest fragments usually work with forests that have been fragmented for decades or just a few hundred years [e.g., 9, 24]. It is therefore possible that most of the studies of anthropogenic forest fragments have failed to record an effect of patch size and isolation on biodiversity because most of these studies have been conducted in recently formed forest patches, where a new equilibrium has not yet been reached. Therefore, the effects of patch size and isolation on the biodiversity of terrestrial fragmented ecosystems can be more effectively assessed in forests that have been fragmented for a long time.

Naturally fragmented forests offer an excellent opportunity for testing the effects of patch size, isolation and other spatial variables on biodiversity because they have been fragmented for thousands or millions of years [25], [26], and consequently can be assumed to have reached equilibrium. The main goal of this study was to assess whether plant species density is explained by patch size and isolation in a naturally fragmented forest on the Yucatan Peninsula. We used species density (number of species in equal-sized samples) as a response variable, as it is a measure of biodiversity that is less influenced by sample area effects than species richness [27], [28]. This forest is characterized by the presence of several forest patches (locally known as Petenes, singular Petén) that are roughly circular, variable in size [25], [29], [30], [31] and grow on Quaternary geological formations ca. 1.7 My old [26], [32]. Scattered near the coast in a wetland matrix [29], [30], [31], the Petenes are landscape units that aptly reflect the habitat patch concept as their spatial boundaries naturally contain or delimit populations and communities of plants [12], [25]. Therefore, the study area allowed us to test the effect of patch area and isolation while controlling for important confounding factors such as patch shape and matrix. In addition to the effects of patch size and isolation we tested the effect of elevation and patch perimeter. Patch elevation is negatively related to the level of salt water during the rainy season and this affects plant distribution [31], [33], while patch perimeter is positively correlated with edge effects [34]. We predicted that plant species density would be positively correlated with patch size and negatively correlated with patch isolation. Patch perimeter would be negatively correlated with plant species density and elevation, positively correlated.

## Materials and Methods

### Study area

**Figure 1:**
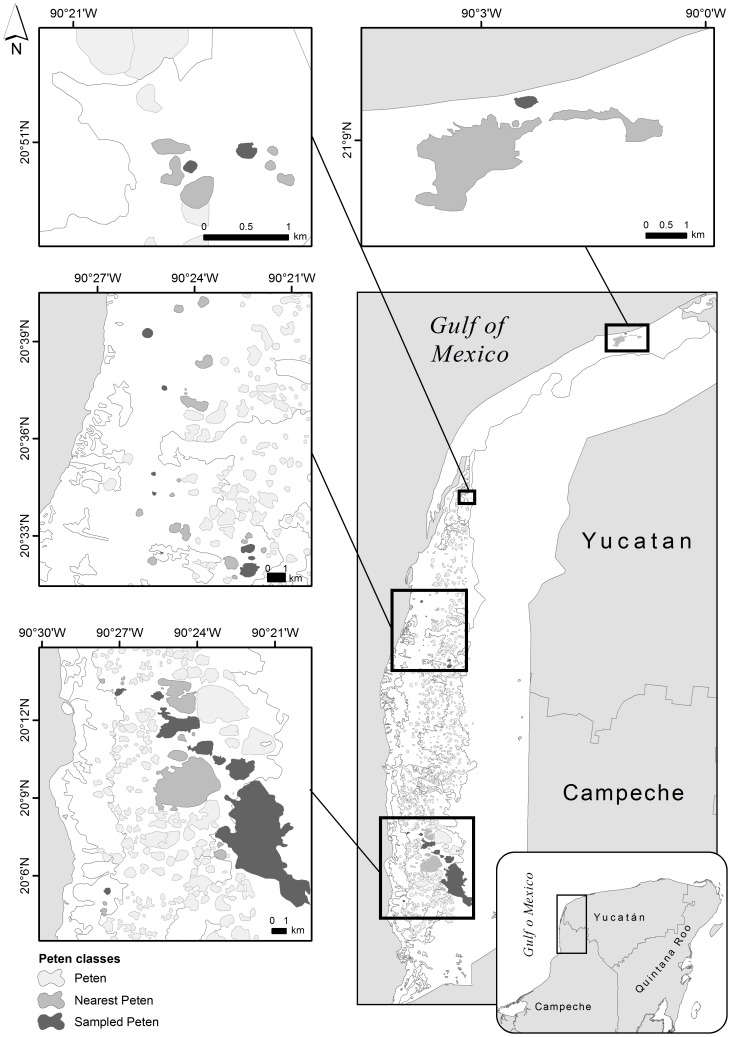
Study area map. Forest patches sampled are in black, and gray patches were used to calculate a connectivity index or the distance to the nearest patch. The white area is the terrestrial portion of the Petenes-Celestún-El Palmar biological corridor. The small rectangle in the inset at the bottom right indicates the position of the study area on the Yucatan Peninsula. All bars represent 1 km.

The study area is the Petenes-Celestún-El Palmar biological corridor (19° 53′–21° 11′ N, 90°28′–90°17′W) located along the northwest coast of the Yucatan Peninsula, which has an area of about 240,000 ha [35] (Fig. 1). The weather is tropical subhumid with summer rains, precipitation is 1000–1200 mm y^−1^ and mean temperature 26.1–27.8°C [36]. Our study focused on naturally formed forest patches of semi-evergreen tropical forest, sometimes mixed with tall mangrove species [30], [31]. These patches are more abundant in a narrow belt (ca.10 km wide) beside the sea and their abundance decreases toward the mainland where the forest becomes continuous (Fig. 1). Most of these forest patches are roughly circular [30], and those that are amorphous are far less frequent [36]. The forest patches grow on Quaternary geological formations that are approximately 1.7 My old [26], [32], and are characterized by taller, more diverse vegetation relative to that of the matrix [30]. It is believed that the presence of these patches is explained by their higher elevation relative to the matrix and the permanent supply of fresh water from one or more sink holes [30]. Tree species such as *Manilkara zapota*, *Metopium brownei*, *Bursera simaruba*, *Laguncularia recemosa* and *Avicennia germinans* dominate the canopy; *Bravaisia tubiflora* and *Sabal yapa* dominate the understorey [26], [31], [37]. The matrix surrounding the forest patches is dominated by short mangrove species (*Rizophora mangle*, *Conocarpus erectus*), sedges (*Eleocharis cellulose*, *Cladium jamaicense*) and cattails (*Typha dominguensis*) [31], [37].

### Sampling

From January 2013 to May 2014 we recorded vascular plants in 19 forest patches using belt transects (50×4 m). It was not possible to select these forest patches randomly owing to insurmountable access difficulties. Epiphytes and lianas were not recorded during the vegetation survey owing to the difficulties associated with assessing their presence and abundance (forest canopy height: 26 m [36]). We recorded woody plants with a girth greater than 5 cm (dbh>1.6 cm) and non woody plants taller than 20 cm in five transects per patch. We did not record small seedlings (dbh≤1.6 cm) because while propagules do arrive in the forest patches, some species cannot establish because of the water level and increased salinity during the rainy season [33]; recording seedlings might have led to an overestimation of real species density. Following the advice of previous studies [9], [27], [28], [38], [39], the area sampled was kept constant in all 19 patches (total sampled area per patch 0.1 ha) to reduce sample area effects. In each patch, the first transect was placed using a random point and the remaining transects were placed systematically: 20 m apart and in a previously defined direction. For all plants, dbh and life form were recorded *in situ*. Plants were identified with the help of field guides [40], [41] and expert advice. Unidentified species were morphotyped (N = 2).

The leaves, flower or fruit of plants of uncertain identity were taken to the laboratory for later identification. Biological material was collected under a permit issued by the Mexican Ministry of the Environment (reg. SEMARNAT 31D8B-00780/1303) and field work was conducted with the permission of the authorities of the protected areas (reg. F00.9.DRBRC.002/13 for Ria Celestún Nature Reserve and reg. F00.9.DRBLP.04/13 for Los Petenes Nature Reserve).

### Spatial configuration

For each patch sampled we measured its area (ha), perimeter (km) and the elevation at its centre (m a.s.l.). As measures of isolation we used distance to the nearest patch (edge to edge in km), distance to the continuous forest (edge to edge in km) and a connectivity index. The latter was an area-based index weighted by distance, calculated as follows:
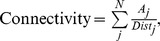
where A is the area of the focal patch (ha) and Dist is the edge to edge distance (km) between the focal patch and another patch in the surroundings. To calculate this index we used the three patches closest to each sampled patch (Fig. 1). For this index, patch connectivity increases as patch isolation decreases (all values >0). All spatial variables were obtained from digital cartography available in Google Earth Pro 7.2. We chose the most recent images available (2009–2011) and verified that the images represented the current landscape in the field. Spatial variables for all 19 forest patches are available in Appendix S1.

### Data analysis

To assess inventory completeness in the patches sampled, we compared the number of species observed in samples to the number of species predicted by three nonparametric estimators: Chao1, ACE and Bootstrap [42]. To minimize potential over- or underestimation of species number by any estimator we used the mean of species predicted by the three estimators as a reference [26]. As previous studies suggest [43], [44], we considered an inventory completeness ≥80% as representative; therefore, only forest patches that met this criterion were included in the analyses (Table 1).

**Table 1:** Number of species observed in 0.1 ha samples (S) and predicted number of plant species using three nonparametric estimators (Chao1, ACE and Bootstrap) for 19 forest patches on the Yucatan Peninsula.

Patch	S	Chao1	ACE	Bootstrap	Average	Completeness (%)
1	6	6±1.3	**7**±0.11	7±0.1	6.7±0.3	90
2	9	10±1.4	10±1.7	10±0.1	10±0	90
3	16	16±0.5	16±21	17±0.1	16.3±0.3	98
4	17	20±2.3	19±1.9	19±1.5	19.3±0.3	88
5	19	19±1.1	20±2.2	22±2.1	20.3±0.9	93
6	16	16±1.9	17±1.2	18±1.9	17±0.6	94
7	19	26±2.4	22±2.1	22±1.7	23.3±1.3	81
8	13	13±1.3	14±1.8	14±0.9	13.7±0.3	95
9	16	16±1.8	18±21	18±1.5	17.3±0.7	92
10	18	20±5.3	23±2.4	20±2.1	21±1	86
11	15	15±3.7	16±1.9	17±1.7	16±0.6	94
12	10	13±2	14±1.8	11±0.9	12.6±0.8	80
13	20	22±5.3	23±2.4	23±2.1	22.7±0.3	88
14	11	12±1.4	13±1.4	12±1.2	12.3±0.3	89
15	11	11±3.7	12±1.6	12±1.3	11.7±0.3	94
16	8	8±0.1	8±1.41	9±0.7	8.3±0.3	96
17	10	10±1.4	10±1.4	10±0.5	10±0	100
18	20	26±2.3	24±2.4	23±3.6	24±1.1	83
19	13	13±1.4	13±1.6	13±0.7	13±0	100

Average is the mean number of species predicted by the three estimators. The percent completeness of our inventory relative to the average predicted number of species is shown (Completeness). In all cases, errors represent one standard error of the mean.

We used plant species density (number of species in 0.1 ha) as the response variable in a model where the predictors were: patch area (ha) on a logarithmic scale, distance to the nearest patch (km), edge to edge distance from the focal patch to the continuous forest (km), patch connectivity index (see “Spatial configuration” above), patch central elevation (m a.s.l.) and patch perimeter (km). This model was fitted to a generalized linear model with a Poisson error distribution and log link function [45]. To determine the minimal adequate model, the complete model was simplified using the AIC criterion and intermediate models were compared using ANOVAs [45] (Table 2). Examination of the residuals of the minimal adequate model indicated a good fit and no evidence of overdispersion (dispersion parameter = 1).

**Table 2:** Log linear models proposed to explain the variation in plant species density (S) in 19 forest patches on the Yucatan Peninsula.

Model	Model description	D^2^	AIC
1	log S = log Size+D. nearest patch+D. continuous forest+Connectivity+Elevation+Perimeter	0.53	110
2	log S = log Size+D. nearest patch+D. continuous forest+Connectivity+Elevation	0.53	108
3	log S = log Size+D. nearest patch+D. continuous forest+Connectivity	0.50	107
4	log S = log Size+D. nearest patch+D. continuous forest	0.47	106
5	log S = log Size+D. nearest patch	0.43	105

Model 1 represents the complete model with six explanatory variables: patch size (Size, on a logarithmic scale), distance to the nearest patch (D. nearest patch), distance to continuous forest (D. continuous forest), patch connectivity index (Connectivity), patch elevation (Elevation) and patch perimeter (Perimeter). Model 5 represents the minimal adequate model and models 2–4 are intermediate steps during model simplification. The Akaike information criterion (AIC) and the proportion of explained deviance for each model (D^2^) are also shown.

## Results

We recorded 55 different plant species in the 19 forest patches sampled (Appendix S2). Species density per forest patch was 6 to 20 species per 0.1 ha (mean = 14±1.44 species; hereafter mean ±1 standard error) (Table 1). The most abundant species in all of the forest patches we sampled was the shrub *Bravaisia tubiflora* (Acanthaceae). The representativeness of our inventory relative to the mean number of species predicted by the three estimators was, in all cases, equal to or greater than 80% (range = 80%–100%; Table 1).

Forest patch size averages 180.15 ha (range: 1.65–2472.82 ha) and average patch perimeter is 4.14 km (range: 0.51–35.21 km), patch altitude is 8.58 m a.s.l. (range: 3–18 m a. s. l.), focal patch to the continuous forest distance is 14.89 km (range: 3.82–89.98 km), focal patch to the nearest patch distance is 3.8 km (range: 0.03–1.62 km ) and the connectivity index is 1794.35 (range: 10.85–12030.91) (Appendix S1).

**Figure 2:**
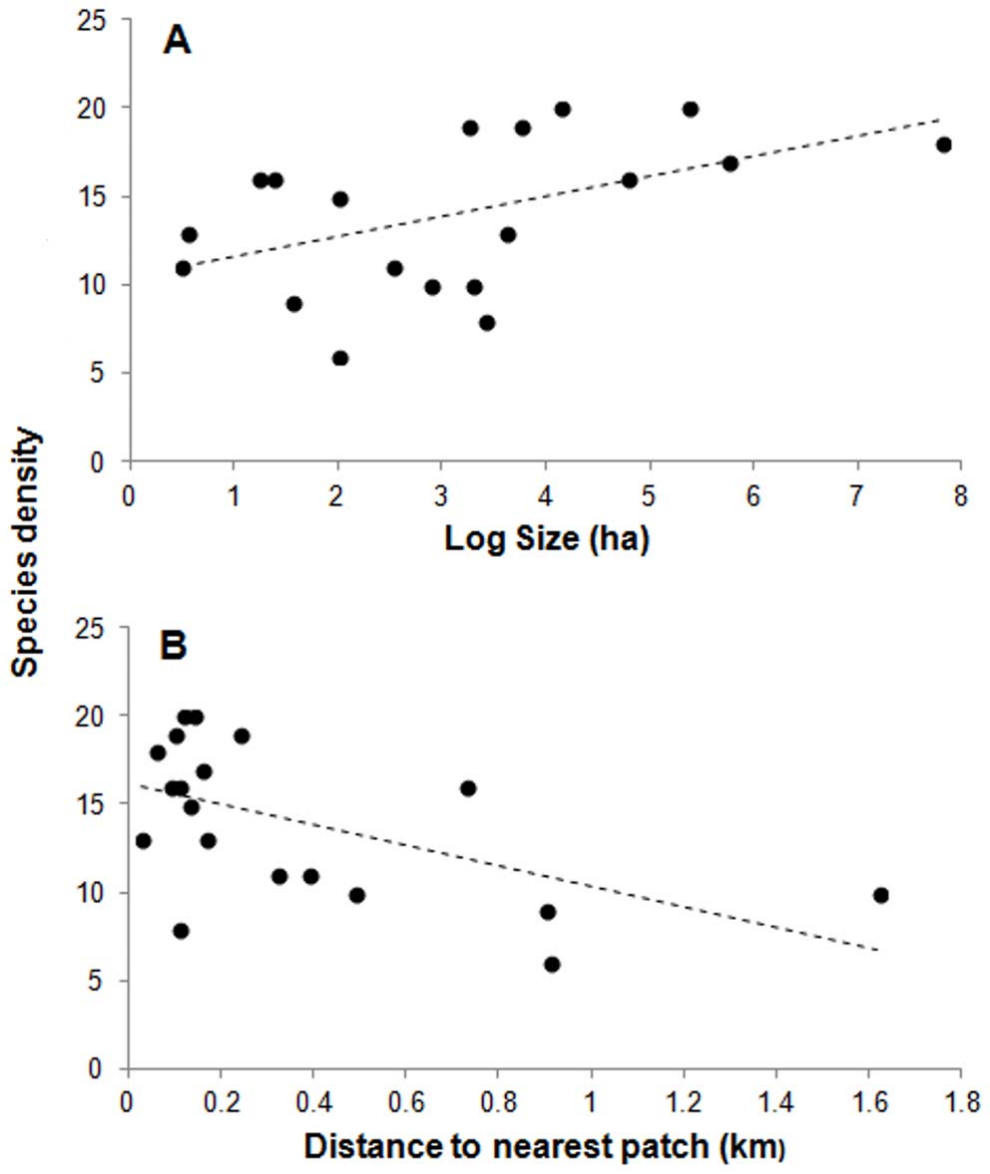
Relationship between plant species density (number of species in 0.1 ha) and patch size (Size) (A) and between plant species density and the distance to the nearest patch (B) for a group of 19 forest patches on the Yucatan Peninsula. Regression lines for A and B are also shown. The scale on the “x” axis for A is logarithmic.

For the model proposed to explain variation in plant species density, of the six variables initially in the complete model, only two (patch size and distance to the nearest patch) were retained in the minimal adequate model (Table 2). The minimal adequate model had the lowest AIC and did not differ statistically from the complete model (χ_4_ = 3.11, *P* = 0.54). The minimal adequate model explained 43% of the total deviance (Table 2). Both of the variables in the minimal adequate model –patch size (χ_1_ = 19.68, *P* = 0.02) and distance to the nearest patch (χ_1_ = 14.78, *P* = 0.03) –significantly affected plant species density. The relationship between plant species density and patch size is positive and logarithmic (y = 1.125 log x +10.501; Fig. 2A), while the relationship between plant species density and the distance to the nearest patch is negative and linear (y = −5.7668×+16.123; Fig. 2B). Patch size and distance to the nearest patch explained 23% and 20% of the deviance of plant species density, respectively.

## Discussion

Although previous studies have obtained contrasting results for the effects of patch size and isolation on species density [e.g. 8, 9, 10], our results clearly show that patch isolation and patch size predict plant species density in our study area on the Yucatán Peninsula. Unlike those of previous studies our study system has been fragmented for a very long time and important confounding factors such as habitat matrix and shape were kept relatively constant in this study. Therefore, we suggest that patch size and isolation are important determinants of species density in fragmented forests, although in some previous studies confounding effects and, more importantly, little elapsed time since fragmentation (i.e. no equilibrium has been reached) have obscured their relevance.

In addition to patch size and isolation, we included other variables in our analysis that were relevant to our study system: patch elevation (negatively correlated with the level of saltwater, which most of the plants in forest patches cannot tolerate; [33]) and patch perimeter (positively correlated with the length of edge in contact with the harsh habitat matrix). However, only patch size and isolation (measured as distance to the nearest patch) significantly explained the variability in species density among forest patches. We believe that patch elevation was not significant because it varies little among patches (3–18 m a.s.l.). Additionally, the internal fresh water supply from the sinkholes that are typically found in petenes [32] may counteract the influence of saltwater from the matrix. Perimeter was probably not a good predictor of species density because most of the patches are roughly circular in shape and therefore variation in area is a better predictor of species density.

In continental islands isolation is clearly the distance to the mainland [1], [2], but it seems that this does not apply to terrestrial ecosystems. In our study system we took distance to the continuous forest to be analogous to the distance to the mainland in oceanic islands. However, this variable did not explain the variation in plant density among patches. We suggest that this is because, unlike oceanic islands where the mainland is the primary species pool for colonization, in most forest fragments immigration occurs predominantly from habitats in the vicinity of the patch, rather than from a common mainland [12]. This notion is reinforced by the fact that the distance to the nearest patch significantly explained variation in species density among patches in our study and in other previous studies [46]. Another metric of patch isolation which was not significant in our analysis was a measure of habitat amount weighted by distance to the focal patch (connectivity index). Therefore, our results do not support the suggestion made in a recent study [12] that habitat amount is a better predictor of the number of species in a focal patch than the distance to the nearest patch is.

The most remarkable result of our study was that patch area and isolation significantly predicted species density. However, we must recognize that the predictive value of these variables is moderate (43%; patch area = 23%, patch isolation = 20%). In a previous study [13], the authors evaluated the predictive power of species-area equations and found that these equations usually explain less than a half (49%) of the variation in species number. Based on this, the authors suggested that predictions by these models are unreliable. We find their position very conservative. Møller and Jennions [47] addressed the question of how much variance can be explained by ecologists. They examined several quantitative reviews of empirical studies and determined that, on average, ecologists can only account for a small portion of variance in their studies (2.5–5.4%) owing to the randomness and noise typical of ecological systems. Therefore, for the results obtained in our study, we think that values of 20 and 23% explained variance are actually quite good. Previous research has suggested that several confounding factors may reduce or nullify the effects of patch size and isolation on species number. For instance, Cook [48] showed that diversity patterns conform better to the prediction of ETIB when matrix species are removed from the patch samples, suggesting that the habitat matrix is creating a confounding effect. However, the results of experimental studies where several confounding effects were controlled also produced inconsistent results with respect to the effects of patch size and isolation on species number [19], suggesting that the time a forest has been fragmented could be even more important than environmental factors in determining the predictive capacity of these variables. Our study system has been fragmented for a period on a scale of geological rather than ecological time and other potential confounding factors were either considered in the model (patch elevation and perimeter) or kept constant (habitat matrix, shape, human disturbance). Therefore, we think that sample size and not being able to choose the forest patches randomly limited the explanatory power of the model.

Another explanation of the contrasting results of previous studies that evaluated the effect of patch size and isolation on biodiversity may lie in how biodiversity was measured. For example, in her review of the effect of habitat fragmentation on biodiversity, Fahrig [7] examined studies where biodiversity was measured as species abundance, species density, species richness, species incidence, genetic diversity, species interactions, species extinction and species turnover. With this kind of variation in the way biodiversity has been measured it is not surprising to find contrasting results and a vague general pattern. In our study we selected the number of species in equal-sized samples (species density) as the response variable. Some authors have preferred species density (obtained from equal-sized samples) over species richness to evaluate species-area relationships [9], [27], [28], [38], [39] because this way patch area is not confounded with the sampled area, as occurs when greater sampling effort is invested in larger patches relative to smaller patches.

The most frequently cited mechanisms to explain the association between species richness and habitat size are sample area effects [49], habitat heterogeneity [50], and immigration-extinction balance [1], [2]. However, in our study some of these mechanisms may not apply because we used species density as a response variable instead species richness. Sample area effects cannot account for the species density-area relationship found in this study because the area sampled was kept constant. As habitat heterogeneity is usually correlated with area [1], [2], habitat heterogeneity is not likely to be the underlying mechanism of this species density-area relationship either. Previous research has suggested that, as occurs with species richness [1], [2], variation in species density can be partially explained by demographic processes such as colonization and extinction [51]. Therefore, we suggest that the most likely mechanism underlying the association between species density and patch area or isolation is a size/isolation dependant equilibrium between colonization and extinction; however, we also recognize that further work is needed to unequivocally identify the mechanism.

In conclusion, species density in fragmented terrestrial ecosystems can be predicted by patch area and isolation. The fact that the study area has been fragmented for a very long period of time may have contributed to this result as this ecosystem has very probably reached equilibrium. We encourage other researchers to test for the effect of patch size and isolation on species number in other naturally fragmented forests to assess whether it has greater predictive power in terrestrial ecosystems that have been fragmented for geological periods of time and are likely to be in equilibrium.

## Supporting Information

Appendix S1 **Spatial configuration (patch size, perimeter, elevation, distance to the continuous forest, distance to the nearest patch, connectivity index) of 19 forest patches on the Yucatan Peninsula, Mexico.**
(CSV)

Appendix S2 **Species abundance in 19 forest patches on the Yucatan Peninsula, Mexico.**
(CSV)
